# Efficacy of Amflow®, a Real-Time-Portable Feedback Device for Delivering Appropriate Ventilation in Critically Ill Patients: A Randomised, Controlled, Cross-Over Simulation Study

**DOI:** 10.1155/2020/5296519

**Published:** 2020-04-24

**Authors:** Jong Won Kim, Sang O Park, Kyeong Ryong Lee, Dae Young Hong, Kwang Je Baek

**Affiliations:** Department of Emergency Medicine, Konkuk University Medical Center, Konkuk University School of Medicine, Seoul, Republic of Korea

## Abstract

**Objective:**

The aim of this study was to test whether Amflow® (a newly designed portable ventilation feedback device) can assist rescuers in delivering target tidal volume (*V*_T_) and respiration rate (RR) during self-inflating bag (SB) ventilations in various clinical scenarios.

**Method:**

This was a simulation study with a prospective cross-over design. A total of 40 trained participants who underwent training for SB ventilation were recruited. Using a SB with or without Amflow® alternately, participants delivered ventilations to test lungs connected to a gas flow analyser in each of three different scenarios: acute respiratory distress syndrome (ARDS; 315–385 ml ranges for 350 ml target *V*_T_, with 20 breaths/min); cardiopulmonary resuscitation (CPR; 450–550 ml ranges for 500 ml target *V*_T_ with 10 breaths/min); and adult head trauma (630–770 ml ranges for 700 ml target *V*_T_ with 15 breaths/min).

**Results:**

The feedback group (SB with Amflow®) demonstrated a significantly higher percentage of delivering the appropriate *V*_T_ ranges than the no-feedback group for both ARDS (58.6% versus 23.5%, respectively) and CPR (85.4% versus 41.0%, respectively) (all *p* < 0.05). However, there was no significant difference between the two groups in the percentage of delivering the appropriate *V*_T_ ranges in head trauma patients (65.9% versus 68.3%, respectively; *p*=0.092). In all three scenarios, a higher percentage of target RR delivered was achieved in the feedback group (88.3%, 99.2%, and 96.3%, respectively) compared with the no-feedback group (5.8%, 12.5%, and 10.0%, respectively) (all *p* < 0.05).

**Conclusion:**

The Amflow® device could be useful for rescuers in delivering SB ventilation with appropriate *V*_T_ and RR simultaneously in various critical situations, except for clinical cases that demand greater delivered *V*_T_.

## 1. Introduction

The self-inflating bag (SB) is a basic device for providing ventilation in critical care management. Positive ventilations can be easily delivered by simply squeezing the bag. However, manual bagging may limit the delivery of appropriate and consistent tidal volume (*V*_T_) because it can vary according to individual factors such as hand size, squeeze power, and technique [1–4]. The rescuers who deliver manual ventilation with a SB may often miss target numbers of ventilations per minute in real-world clinical settings if they are unable to continuously concentrate on the procedure. Excessively high respiration rate (RR) during cardiopulmonary resuscitation (CPR) has been well documented, and avoiding hyperventilation is a crucial element during CPR [5, 6].

Education programs and training may be helpful in advancing SB skills [7]. However, this is time consuming. Chaotic and emergent situations, such as cardiac arrest, may disturb the delivery of accurate ventilation in the real world [6, 8]. Alternatively, some researchers have suggested that the use of a feedback or a monitoring device may be useful in delivering correct RR [9, 10]. Devices designed to guide the delivery of correct *V*_T_ have also been introduced in simulation settings [11–14]. However, to our knowledge, there were no measures that controlled the delivery of ventilations with correct *V*_T_ and RR simultaneously.

Amflow® (MEDICION, Goyang, Korea) is a newly developed medical device for monitoring SB ventilation. It displays real-time measures of timing to provide ventilation and deliver *V*_T_ on a screen simultaneously, which enables the rescuer to deliver target *V*_T_ and RR simultaneously. We hypothesized that Amflow® could be helpful for rescuers in delivering a higher percentage of target *V*_T_ and RR in various clinical situations. The aim of this study was to test whether the Amflow® device could assist rescuers in delivering target *V*_T_ and RR simultaneously during SB ventilations in various simulated clinical settings.

## 2. Methods

### 2.1. Study Design and Subjects

This study, which was performed in a simulated setting using an artificial lung and a testing device, had a prospective, cross-over, and randomly assigned execution design. The protocol was approved by the Institutional Review Board for Human Research at Konkuk University Hospital, Seoul, Korea (KUH 1260022). Study participants were recruited from among a pool of senior medical and emergency medical technician students. The minimal sample size required was calculated based on the proportion of correct ventilations. Before the study, a pilot simulation test was performed to predict estimated proportion values in the intervention and control groups (75% versus 40%). For an alpha error of 5% (two-sided) and a power of 80% in the randomized study between two groups, the estimated sample size required was 37. Using a projected drop-out rate of 10%, 40 subjects were enrolled.

### 2.2. Study Device

**Figure 1:**
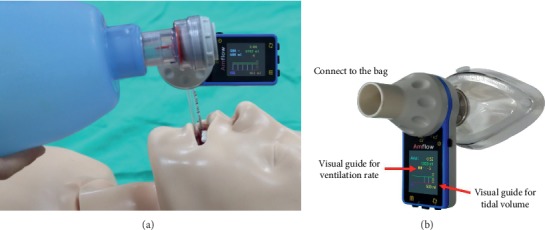
Photograph of Amflow® (MEDISION CO, Seoul, Korea). It can be easily placed at the bag-mask and the endotracheal tube, and then the rescuer can deliver the accurate ventilation rate and tidal volume by live feedback using the screen of Amflow® (a). Its screen can supply the alarm of ventilation rate and show the changes of tidal volume via the bar graph (b).

Amflow® is a live feedback device for ventilation, which was developed by MEDICION and approved by the Korea Food and Drug Safety Administration. It can be situated between a SB and facial mask or endotracheal tube (Figure 1). Air flow generated by squeezing the bag passes through the pipe of the device, which in turn rotates a turbine. An infrared sensor in the pipe measures the number of turbine rotations, and *V*_T_ is calculated based on turbine revolutions and velocity. *V*_T_ measured can be displayed on a screen using a bar graph. When a rescuer begins squeezing the bag, a *V*_T_ bar on the screen increases gradually. The rescuer can stop squeezing the bag when the *V*_T_ bar reaches a preset line representing the target *V*_T_ on the screen. In addition, a countdown timer is displayed to assist in the delivery of target RR. Therefore, the device enables rescuers to control the accuracy of *V*_T_ and RR simultaneously by monitoring the screen display.

### 2.3. Education and Testing Process

After an explanation of the purpose of the study, subjects provided informed written consent for participation. Before commencement of the study, all participants attended a SB ventilation training session with and without the Amflow® device. The training session consisted of a lecture describing the device, a review of ventilation skills, and a 30 min practice session. When participants practiced the bagging themselves with and without Amflow® alternately, they were able to check *V*_T_ and RR by viewing the ventilation data from the gas flow analyser (VT PLUS HF, Fluke Biomedical, Everett, WA, USA), which was connected to the self-inflating bag. All participants underwent sufficient training for delivering various target *V*_T_ (350, 500, and 700 ml) and *V*_T_ (10, 15, and 20 breaths/min).

**Figure 2:**
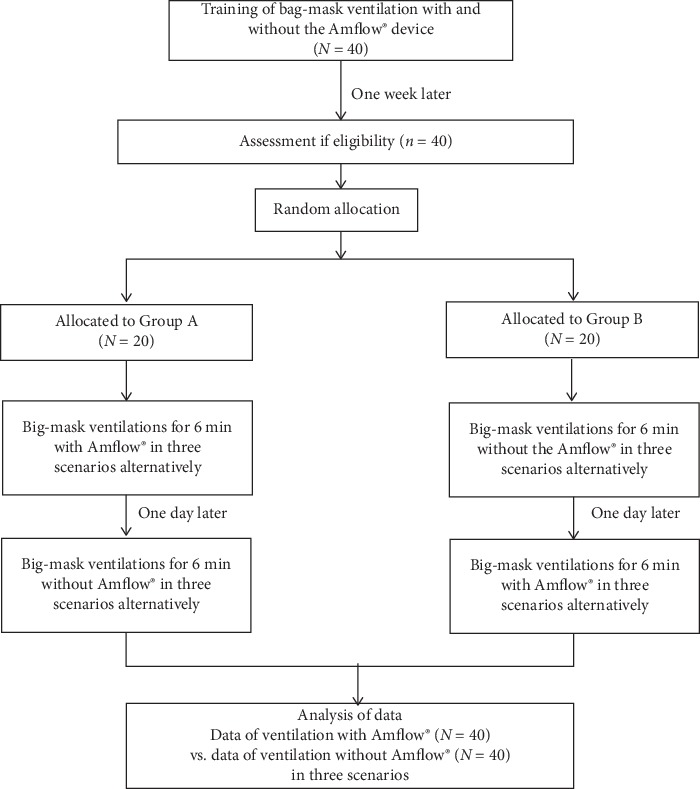
Flowchart of the study.

One week after completion of the ventilation training session, participants were recalled to the simulation centre for the study. The participants were randomly assigned to group A or B using the sealed enveloped selection method. Participants were asked to provide ventilations for 6 min in each of the three following scenarios: a patient with acute respiratory distress syndrome (ARDS; 350 ml of target *V*_T_ at 20 breaths/min) [15, 16]; cardiopulmonary resuscitation (CPR; 500 ml target *V*_T_ at 10 breaths/min) [17]; and a head trauma patient with the normal lung requiring ventilator support (700 ml target *V*_T_ at 15 breaths/min [18]. We tried to vary the order of scenarios to minimize any learning bias. For three scenarios, total six orders were available, and total 24 envelops (four envelops each order) were prepared. The study participants performed the ventilations according to the order of envelop chosen by him/herself. We had the participants squeeze the SB with one hand. After each trial, up to 10 minutes of rest was allowed in order to reduce the participant's fatigue. Participants in group A performed the three scenarios under real-time Amflow® feedback, whereas those in group B performed the three scenarios without Amflow® assistance. After each of the protocols was completed, each group exchanged their study protocol (i.e., cross-over) and performed the three scenarios again after a sufficient break (1 day) (Figure 2).

**Figure 3:**
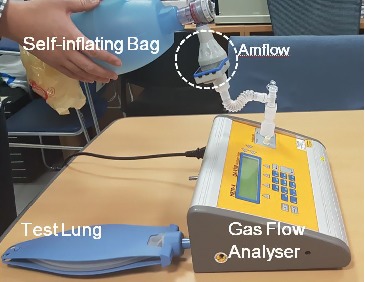
Photograph of the assembly including self-inflating bag, Amflow® (feedback device), tube, and gas flow analyser which is connected with the artificial lung.

During the test, SB was connected to the gas flow analyser (Figure 3). A test lung was connected to the other end of the device. Amflow® was connected between the SB and the gas flow analyser through a tube. All *V*_T_ were displayed on the screen of the gas analyser. We recorded the data displayed on the screen using a camcorder (Samsung, Seoul, Korea), and values of *V*_T_ were collected and RRs were counted by reviewing the recorded video clips.

### 2.4. Outcomes and Analysis

The primary outcome measure of the study was the percentage of appropriate *V*_T_ which was within 10% range of the target volume: (1) 315–385 ml for 350 ml target, (2) 450–550 ml for 500 ml target, and (3) 630–770 ml for 700 ml target. The secondary outcomes were appropriate RR when the participants deliver the exact number of target ventilations per minute. For comparing categorical variables, the chi-squared test was used. For comparing continuous variables, we used the paired *t*-test if variables were normally distributed. If continuous variables were not normally distributed, the Wilcoxon signed-rank test was used. Data were analysed using SPSS version 15.0 (IBM Corporation, Chicago, IL, USA); differences with *p* < 0.05 were considered to be statistically significant. Distribution bar plots were used to illustrate differences in *V*_T_ and *V*_T_ between Amflow®-assisted and conventional ventilation.

## 3. Results

### 3.1. Baseline Data of the Study

A total of 40 participants (26 to 35 years of age and 37 (92.4%) were males) were enrolled in this study.

### 3.2. Delivery of Appropriate *V*_T_

In the ARDS (315–385 ml), CPR (450–550 ml), and head trauma (630–770 ml) scenarios, the mean delivered *V*_T_ was 361.14 ± 34.09 ml, 505.56 ± 32.21 ml, and 656.64 ± 60.37 ml, respectively, in the feedback group. The no-feedback group delivered higher mean *V*_T_ in all three scenarios (all *p* < 0.05) (Table 1).

**Figure 4:**
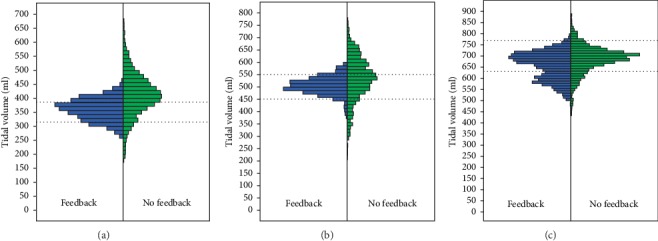
Distribution bar plots for tidal volumes (ml) between the feedback group (bag-mask ventilation by using Amflow®) and the no-feedback group in three scenarios. (a) ARDS scenario (315–385 ml). (b) Cardiac arrest scenario (450–550 ml). (c) Head trauma scenario (630–770 ml).

In the ARDS scenario, a higher percentage of appropriate *V*_T_ delivered was observed in the feedback group compared with the no-feedback group (58.6% versus 23.5%, respectively; *p* < 0.001). The feedback group demonstrated a higher percentage of appropriate *V*_T_ for cardiac arrest patients compared with the no-feedback group (85.4% versus 41.0%, respectively; *p* < 0.001). However, there was no significant difference between the two groups in the percentage of appropriate *V*_T_ delivered for a head trauma patient (65.9% versus 68.3%, respectively; *p*=0.092). Different distribution patterns between the two groups are shown in Figure 4. Compared with the feedback group, the no-feedback group demonstrated a more varied distribution of *V*_T_ and higher frequency of delivery of *V*_T_ that were out of the appropriate *V*_T_ range in the ARDS and CPR scenarios.

### 3.3. Delivery of Appropriate RR

In the ARDS (20 breaths/min), cardiac arrest (10 breaths/min), and head trauma (15 breaths/min) scenarios, the median RR were 20 (interquartile range (IQR, 25^th^ percentile–75^th^ percentile)) 20–20), 10 (IQR 10–10), and 15 (IQR 15–15) breaths/min, respectively, in the feedback group. The median RR in the no-feedback group was 16.9 (IQR 11.6–19.2), 9.4 (IQR 8.2–12.2), and 13.8 (IQR 11.4–16.8) breaths/min in the three scenarios, respectively. A significant difference was observed only in the ARDS scenario (*p* < 0.001).

**Figure 5:**
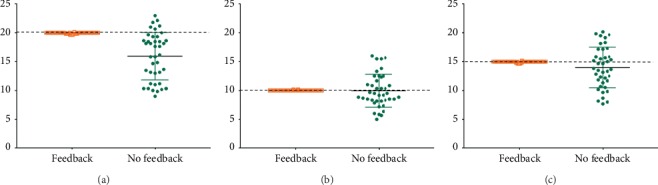
Dot plots for respiration rate (breaths/min) between the feedback group (bag-mask ventilation by using Amflow®) and the no-feedback group in three scenarios. (a) ARDS scenario (20 breaths/min). (b) Cardiac arrest scenario (10 breaths/min). (c) Head trauma scenario (15 breaths/min).

In all three scenarios, including appropriate RR ranges of 20, 10, and 15 breaths/min, a higher percentage of appropriate RR delivered was achieved in the feedback group (88.3%, 99.2%, and 96.3%, respectively). The no-feedback group demonstrated a significantly lower percentage of appropriate RR delivered in all three scenarios (5.8%, 12.5%, and 10.0%, respectively) and a wide distribution of RR in all three scenarios (Figure 5).

## 4. Discussion

In the present simulation study, we attempted to demonstrate the superiority of SB ventilation with the feedback using the Amflow® device over conventional ventilation (i.e., no-feedback) in various simulated scenarios. Use of the feedback device demonstrated an overall benefit of delivering appropriate *V*_T_ and RR. For the delivery of target RR, Amflow® exhibited significantly greater accuracy (≥90%) compared with the no-feedback group, which had very low accuracy (≤10%). For the delivery of appropriate *V*_T_, the feedback group could also deliver a higher percentage of appropriate *V*_T_ in the ARDS (315–385 ml) and CPR (450–550 ml) scenarios. However, this feedback may have a limitation in delivering greater *V*_T_ ranges (630–770 ml) in high-demand situations.

Appropriate ventilation is a crucial component in emergent situations [19]. According to the patient conditions, such as ARDS, trauma, and cardiac arrest, different target *V*_T_ or RR are demanded from rescuers who are responsible for squeezing the SB. Inappropriate SB ventilations can be seriously harmful in some situations, the most widely known of which is hyperventilation during CPR [5, 6]. Excessive ventilations may cause a decrease in the cardiac output by increasing intrathoracic pressure in most critically ill patients who are treated by positive ventilations. Excessive *V*_T_ may also lead to gastric inflation and subsequent complications. In contrast, excessively low ventilations may aggravate hypoxia or hypercapnia due to limited oxygen supply or carbon dioxide elimination. Therefore, careful control of positive ventilations has been accepted as an important factor during CPR [17]. The first concern started from controlling RR during CPR because it could be easily implemented and cost effective. Audible feedback can easily monitor the squeeze timing of the bag using a simple timing device [9, 10]. The use of feedback for correct RR has often demonstrated dramatic efficacy; in contrast, however, most cases without the feedback have demonstrated significantly lower accuracy of RR [6, 20]. For nonarrest patients, this may be important when they are ventilated using a SB before mechanical ventilation or are transported temporarily in hospital or out-of-hospital settings.

Feedback for delivering appropriate *V*_T_ is not easy due to inherent technical difficulties. *V*_T_ can be easily varied according to individual factors, such as the manual technique used for squeezing the bag (i.e., one-handed or two-handed), hand size, grip speed, or power [1, 2, 4, 21]. Numerous studies have reported that many rescuers do not deliver correct *V*_T_ regularly in simulation settings [11–13, 21, 22]. To overcome this problem, two approaches have been used to consistently guide the delivery of target *V*_T_ ranges. Some researchers modified the SB by marking the SB for correct delivery of *V*_T_ [12–14]. Their hypothesis was that steady compression at a specific point on the bag can lead to consistent delivery of target *V*_T_ regardless of variations in personal characteristics. Their simulation studies demonstrated that modified SBs were superior than conventional SB for delivering correct VT. Another modification of the SB was to integrate a solid internal handle situated inside the bag, such as in the Spur II BVM device (Ambu, Ballerup, Denmark) [23]. This can support downward pressure for sufficient *V*_T_ delivery when the rescuer squeezes the bag using a one-handed technique. This modified bag yielded higher *V*_T_ compared with the conventional SB. The second approach was to connect an external feedback device to SB. You et al. developed a real-time *V*_T_ monitoring device, which can be situated between the inlet of the bag-valve and the outlet of the endotracheal tube [11]. This device can provide actual numerical *V*_T_ data using information from Hall-effect sensors and the time of open airway during SB ventilation. In a simulated cardiac-arrest setting, the feedback group using this device delivered a higher percentage of correct *V*_T_. However, all of the aforementioned methods focused only on delivering correct *V*_T_ and do not include the simultaneous feedback for RR. In addition, previous simulation studies were confined only to the cardiac-arrest situation; as such, other critical situations that demand different *V*_T_ and RR were not investigated.

Amflow® has a setting method similar to the real-time *V*_T_ monitoring device designed by You et al. The difference is that volumes were calculated from the flow of the internal turbine. Benefits of Amflow® are that it supports real-time guidance of target *V*_T_ and RR simultaneously via screen indicators. Therefore, rescuers could deliver accurate minute ventilations to the patients with Amflow® compared to other feedback devices, which provide only either *V*_T_ or RR. In addition, its presetting function can enable easy adjustment of target *V*_T_ and RR according to the patient's lung condition, disease, weight, and various other factors. To the best of our knowledge, this device may be the first portable ventilation feedback device enabling simultaneous control of *V*_T_ and RR. Amflow® has the advantage of providing feedback by visual signals because it is difficult to receive audible feedback given the often distracted concentration of the rescuer due to the surrounding noise in emergent situations. In addition, the ventilation feedback should be portable, provide real-time monitoring, and be easy to apply. We believe that Amflow® is a good feedback device in terms of providing these features.

Different from previous simulation studies that included only CPR situations, we challenged the accuracy of Amflow® in various clinical conditions in which various target *V*_T_ and RR are required. We evaluated three different situations with various *V*_T_ and RR. In this study, Amflow®-assisted ventilation demonstrated statistically significant higher accuracy for targeting RR compared with conventional ventilation (i.e., no feedback). This result is consistent with previous studies in which correct RR with less variance was delivered with audible guidance. Despite the emphasis on appropriate RR, many participants exhibited a wide distribution pattern of RR delivered when the feedback was not provided.

For delivering appropriate *V*_T_, Amflow® can assist in achieving delivery of a high percentage of appropriate *V*_T_ in the ARDS (315–385 ml) and the CPR (450–550 ml) scenario. However, the feedback device did not guarantee a higher percentage of delivering appropriate *V*_T_ in the scenario demanding large *V*_T_ ranges (630–770 ml). As feedback of *V*_T_ via elevation of a screen bar takes a relatively long time due to more turbine rotation, the rescuer may experience difficulty with receiving feedback based on a slowly increasing bar graph on the screen to match *V*_T_ every 4 s. Second, our study permitted only the one-handed bag compression technique because it included only cases of a lone rescuer who should hold the mask using one hand and compress the bag with the other. Compared with the two-handed compression technique (approximate *V*_T_ range, 500–800 ml), the one-handed compression technique resulted in generally lower ranges of *V*_T_ (approximately 400–700 ml) when the bag was compressed manually without the feedback [3, 21]. For rescuers with small hands, the one-handed technique may be limited in generating sufficient *V*_T_. This pattern was clearly reflected in the distribution figure of the head trauma scenario regardless of the usage of a feedback device.

## 5. Study Limitations

Our study had several limitations, the first of which were those inherent to simulation studies. It could not reflect normal respiratory physiology such as airway resistance or lung compliance in the patients. Some cases that can increase resistance at the outlet point of the device (e-tube folding or severe upper airway obstruction) can hinder turbine rotation which supplies the volume data. Therefore, the insufflated volume of the manual ventilation with the SB may not match actual *V*_T_ in these situations. It also could not replicate the stressful conditions of the prehospital field or the emergency room. As such, it may have different results in real-world situations, and we cannot be sure whether our results are generalizable. Second, as the SB is squeezed by hands of rescuers, personal factors such as hand size, volume, and grip power may affect *V*_T_ delivered. Its effect may be different according to the diverse scenarios, but we could not control this confounding factor. Third, we evaluated ventilation in a period of only 6 min. Our participants complained of fatigue because they had to continuously monitor during Amflow®-assisted ventilation. Rescuers may experience more fatigue than our subjects, and different results may have occurred if they had to perform manual ventilation for a longer time. Fourth, we performed our study by directly connecting the SB to a gas flow analyser. The SB could also be connected to a facial mask in a prehospital field. The issue of mask leak due to incomplete sealing is inevitable when manual ventilation is performed using a facial mask; as such, it is necessary to consider this factor. Fifth, we recruited the participants among medical and emergency medical technician students. They were inexperienced rescuers, and our result might be different with highly trained rescuers.

## 6. Conclusion

Feedback using the Amflow® device was helpful for rescuers in delivering SB ventilation with appropriate *V*_T_ and RR simultaneously in various critical situations, except in clinical scenarios that demanded the delivery of large *V*_T_.

## Figures and Tables

**Table 1:** Comparable data of ventilations between the feedback (using Amflow®) and the no-feedback group.

Scenario	Parameters	Feedback	No feedback	*p* value
Adult respiratory distress syndrome (350 ml, 20/min)	Tidal volume (ml); mean ± SD	361.14 ± 34.09	412.57 ± 67.07	<0.001
	Frequency of accurate volume range; no. (%)	2806/4789 (58.6)	895/3807 (23.5)	<0.001
	Respiration rate per min; median (25%, 75%)	20 (20, 20)	16.9 (11.6, 19.2)	<0.001
	Frequency of accurate rate; no. (%)	212/240 (88.3)	14/240 (5.8)	<0.001

Cardiopulmonary resuscitation (500 ml, 10/min)	Tidal volume (ml); mean ± SD	505.56 ± 32.21	534.15 ± 73.54	0.012
	Frequency of accurate volume range; no. (%)	2052/2402 (85.4)	975/2380 (41.0)	<0.001
	Respiration rate per min; median (25%, 75%)	10 (10, 10)	9.4 (8.2, 12.2)	0.619
	Frequency of accurate rate; no. (%)	238/240 (99.2)	30/240 (12.5)	<0.001

Head trauma with the normal lung (700 ml, 15/min)	Tidal volume (ml); mean ± SD	656.64 ± 60.37	684.88 ± 53.04	0.013
	Frequency of accurate volume range; no. (%)	2368/3593 (65.9)	2296/3361 (68.3)	0.092
	Respiration rate per min; median (25%, 75%)	15 (15, 15)	13.8 (11.4, 16.8)	0.104
	Frequency of accurate rate; no. (%)	231/240 (96.3)	24/240 (10)	<0.001

## Data Availability

The data used to support the findings of this study are available from the corresponding author upon request.
